# Challenges Perceived by Health Care Providers for Implementation of Contact Screening and Isoniazid Chemoprophylaxis in Karnataka, India

**DOI:** 10.3390/tropicalmed6030167

**Published:** 2021-09-14

**Authors:** Kiran Chawla, Sharath Burugina Nagaraja, Nayana Siddalingaiah, Chidananda Sanju, Vishnu Prasad Shenoy, Uday Kumar, Arundathi Das, Druti Hazra, Suresh Shastri, Anil Singarajipur, Ramesh Chandra Reddy

**Affiliations:** 1 Department of Microbiology, Kasturba Medical College Manipal, Manipal Academy of Higher Education (MAHE), Manipal 576104, India; vishnu.shenoy@manipal.edu (V.P.S.); drutihazra@gmail.com (D.H.); 2 Employees State Insurance Corporation Medical College and Post Graduate Institute of Medical Sciences and Research, Bengaluru 560010, India; sharathbn@yahoo.com (S.B.N.); uday0908@gmail.com (U.K.); 3 District Tuberculosis Office, Udupi 576101, India; sanjuchida@rediffmail.com; 4 District Tuberculosis Office, Bengaluru Bruhat Mahanagara Pallike (BBMP), Bengaluru 560011, India; dtokablc@rntcp.org; 5 State Tuberculosis Office, Bengaluru 560027, India; susha007@gmail.com (S.S.); dadranil@gmail.com (A.S.); vrcreddy@live.com (R.C.R.)

**Keywords:** challenges, healthcare providers, pediatric tuberculosis, India

## Abstract

**Background:** In India, challenges in pediatric TB contact screening and chemoprophylaxis initiation are still underexplored. Elucidating these challenges will help in better implementation of the programme at the grass-roots level thereby helping in early detection of pediatric cases and timely initiation of preventive therapy. This study aimed at exploring the challenges faced by the health care provider in contact screening and chemoprophylaxis initiation implementation of the pediatric household contacts. **Methods:** A qualitative study was conducted in the districts of Bengaluru and Udupi and in-depth interviews of key participants were adopted to explore the challenges. Qualitative data analysis was done after developing transcripts by generating themes and codes. **Results:** The key challenges were identified as stigma towards the disease, migrant patients with changing address, difficulty in sample collection, anxiety among parents due to long duration of the prophylactic treatment and adherence to IPT is not well documented, inadequate transportation from rural areas, and the ongoing COVID-19 pandemic. **Conclusions:** It is important for the National TB programme to address these challenges efficiently and effectively. Innovative solutions, feasible engagements, and massive efforts are to be taken by the programme to improve contact screening and isoniazid chemoprophylaxis implementation.

## 1. Introduction

Pediatric tuberculosis (TB) is one of the major causes of morbidity and mortality in children below 15 years of age and accounts for 12% of TB incidence (1.2 million out of 10 million TB cases). India shares a major part of the 31% global burden for pediatric TB cases [1,2]. Pediatric TB is often considered difficult to diagnose and treat. The TB incidence is high amongst young children (<6 years) as they are easily prone to contract infections. Children are more likely to end up suffering from serious forms of the disease such as Tubercular meningitis. The National Tuberculosis Elimination programme (NTEP) in India recommends household contact screening to identify children with the disease and to start with isoniazid preventive therapy (IPT) for contacts [3,4]. With increasing rates of TB cases in children, there lies a potential to improve the implementation of systemic contact screening [2,3]. However, contact screening and isoniazid preventive therapy (IPT) initiation are faced with several challenges such as adult patients not being informed about the screening, patient reluctance, etc. [5,6,7]. It becomes very important to understand the lacunae in the implementation of the programme.

Pediatric household contacts are defined as children less than 6 years of age living in the same household as or in frequent contact with a source case (e.g., the child’s caregiver) with multi-drug resistant tuberculosis (MDR-TB) or one who shared the same enclosed living space for one or more nights or frequent or extended periods during the day with the index case during the 3 months before the commencement of the current treatment episode.

A scoping review from Campbell et al. highlighted the fact that a well-defined cascade of care is not being followed for systematic evaluation of pediatric latent TB infection. Most of the studies have focused on the aspect of treatment initiation and completion, while the other components such as screening, investigation, and not initiating TB preventive treatment are given lesser importance [8].

A qualitative study conducted in Lesotho on facilitators and challenges in childhood TB prevention revealed that the key challenges were difficulties in access to care, supply-chain management issues, identification and screening of child contacts, and optimal adherence to isoniazid preventive therapy [9].

Based on the study conducted by Madhavi et al., it is estimated that for every 100-smear positive TB patient there would be at least 20 child contacts less than 6 years who are eligible for IPT [10].

In the year 2020, the Bengaluru Urban district had notified 11,165 TB patients (6830 public sectors, 4335 private sectors) [11]. We presume that 50% of the cases notified from the public sector would be microbiologically confirmed cases and based on the estimates there would be 689 eligible children for IPT for patients notified under the public sector. Similarly, the Udupi district had notified 1190 TB patients (837 public sectors, 353 private sectors) and there would have been 83 eligible children for IPT for patients from the public sector. However, under the programme, there are no established reporting mechanisms to monitor and ensure that all the eligible children from the public sector were initiated on IPT and there was no information on the eligible children of TB patients notified from the private sector. Moreover, among those initiated on IPT, there was no systematic supervision and monitoring of chemoprophylaxis from the health system. There is a wide gap between those children found eligible and those initiated on the treatment. Although the programme has developed standard guidelines to screen child contacts, the activity has seldomly got prioritized and implemented in great vigor and dedication in the field due to many operational issues.

We conducted this study to understand the operational challenges faced by health care providers to identify the child contacts and initiate them on IPT and to suggest pragmatic solutions to overcome the challenges. The healthcare staffs work closely with the patients and hence they can reflect the challenges and ground realities, to render these services to the patient and their family members [1,2,12].

## 2. Materials and Methods

### 2.1. Study Design

A qualitative exploratory study design was adapted to understand the perceptions of health care providers on isoniazid prophylaxis implementation from February 2021 to June 2021.

### 2.2. Study Setting

**Figure 1:**
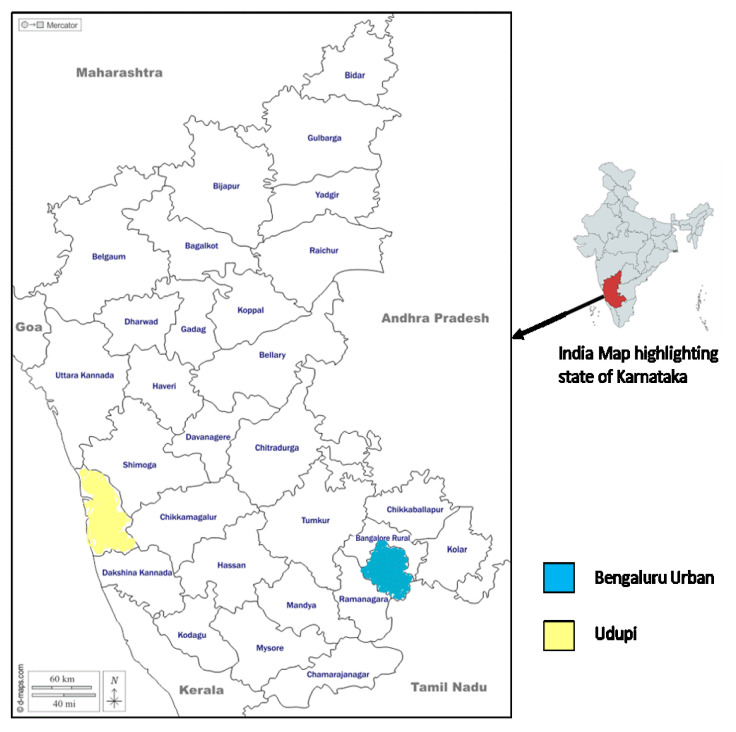
The highlighted areas are the selected study sites i.e., Bengaluru urban and Udupi districts in Karnataka.

Karnataka is one of the south Indian states with a population of 61.1 million (2011 data) with a total of 31 administrative districts [13]. We chose to conduct the study at the Bengaluru urban and Udupi districts of Karnataka State (Figure 1).

Bengaluru Urban—Bengaluru is the capital and largest city of Karnataka and has a population of over 8.5 million (2011 data) [13]. Being a thickly populated metropolitan city and having people from different parts of the state, the population is diverse. The health care services are mainly provided by both the public and private sectors. The public health services are provided through the Bruhat Bengaluru Mahanagara Palike (BBMP) corporation. The public sector comprises 204 urban primary health centers, three government medical colleges, and three tertiary care hospitals. The private sector comprises 67 corporate hospitals, 814 nursing homes, and 2433 private practitioners’ clinics.

Udupi—Udupi is part of coastal Karnataka with a 1.17 million (2011 data) population, while Udupi is a small town but known to have high literacy rates [13]. The district is sub-divided into six tuberculosis units (TU). There are 61 primary health centers, six community health centers, two sub-district hospitals, and one medical college. The district has eight government pediatricians and nine X-ray units under the public sector.

The two districts were chosen to understand the various challenges faced by healthcare providers in different and unique settings. Both districts have good health infrastructure in terms of health facilities and human resources.

### 2.3. Study Population

The healthcare staff workers involved in delivering the services were included in the study. In addition, the NTEP programme staff and the general health system were part of the study. The district TB officer (DTO) manages the overall NTEP at the district level. At the sub-district level, the TB unit includes designations such as senior treatment supervisor (STS), senior TB laboratory supervisor (STLS), and TB health visitor (TB-HV) at urban centers, as well as the pediatrician who works towards the diagnosis, initiation of treatment, treatment adherence, and obtaining a positive outcome. At Peripheral Health Centers, the medical officer (MO), auxiliary nurse midwife (ANM), and an accredited social health activist (ASHA) are responsible for public health actions such as implementing various health programmes, contact tracing, managing co-morbidities, and direct bank transfers to the patient’s account. Interviews were conducted with staff at all the designative levels to obtain varied perceptions of the challenges.

### 2.4. Data Collection

In-depth, face-to-face interviews were conducted by the trained researcher using a pre-tested interview guide in the local language (Kannada) or English as deemed appropriate. All the interviews were audio-recorded using a mobile voice recorder after obtaining written informed consent from the participant. The interviews were conducted at a place and time convenient to the respondents. It was ensured that the place of the interview was quiet, calm, and the respondents were made comfortable. Each of the interviews was designed to last for 30 min. The interviewers checked their understanding of the key points of respondents by summarizing the interview at the end.

### 2.5. Data Analysis

The interview recordings were transcribed and then analyzed using thematic analysis as a method, then scrutinized line-by-line to identify few recurring phrases and labeled as codes by the trained researcher [14]. Verbatim quotes were selected from the interviews supporting the codes selected. The analysis was reviewed by the principal investigator and co-investigators to increase the credibility. To ensure the confidentiality of the study participant, we mentioned the designation and district of the healthcare providers.

### 2.6. Ethics Approval

Ethical approval was obtained from the Institutional Ethics Committee of both Kasturba Medical College, Manipal (626/2020) and ESIC Medical College and PGIMSR, Bengaluru (523/L//11/12/Ethics/ESIC Medical College and PGIMSR, Bengaluru, India). An approval letter was obtained from the state TB officer to conduct the study at the respective sites. Permission was obtained from the district TB officer, Bengaluru, and Udupi to conduct the interviews. Informed written consent was obtained from all the participants.

## 3. Results

The interviews lasted an average of 39 min (range, 7–68 min). A total of 64 interviews were conducted, with 33 interviews in Bengaluru of which eight STS, eight TBHV, and 16 general health staff and DTO participated. In addition, 31 interviews were conducted in Udupi of which six STS, three TBHV, four medical officers, four pediatricians, and 13 general health staff and DTO participated in the study.

### 3.1. Challenges

**Figure 2:**
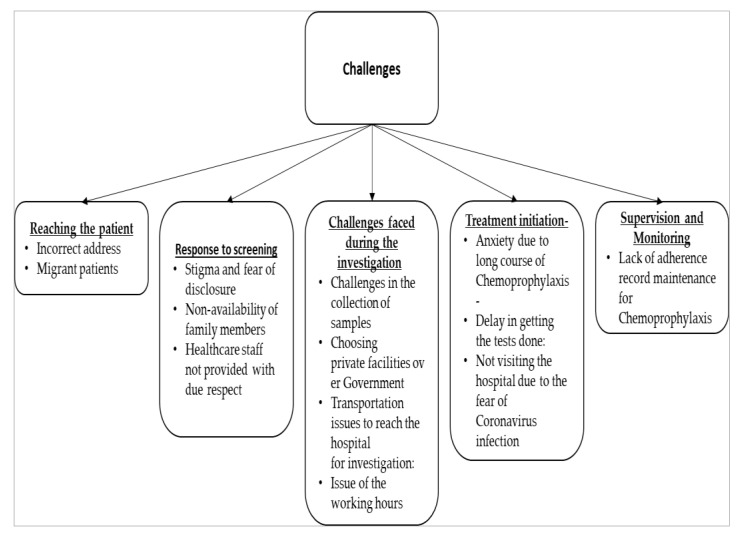
Depicting the key challenges faced by the healthcare staff.

The details of the challenges are enumerated under the following subheadings and the key challenges faced by the healthcare providers are shown in Figure 2.

#### 3.1.1. Reaching the Patient

**1.**  ***Incorrect address:*** The health care providers expressed those patients belonging to other districts provided the wrong address and contact number as they did not wish to be traced back and were hesitant to let their family members know about the disease. Although the percentage of people could be small, it still contributes to patients who are lost to follow-up. Some patients refrain from sharing it with their families as they believe that the family might not be supportive. The pediatric contacts from this kind of patient’s household will be missed for screening and evaluation.

*“Wrong address and mobile numbers are given by daily wage workers particularly from districts like Koppala, Raichur, and Gadag…”*

(STS, Udupi district)

*“It becomes difficult to reach the patient’s address. One of the patients had given us the wrong address. Later STS called them here to the hospital and that is when I got to know that the patient lives in my area*”

(ASHA, Bengaluru district)

**2.**  ***Migrant patients:*** Many healthcare providers explained that most of the migrant population are from the northern part of the state who travel from place to place for their livelihood. Managing and ensuring treatment adherence becomes challenging when they frequently shift cities. The capital of Karnataka state, Bengaluru, is home to several lakhs of migrant workers who change their homes based on work. In addition, tracking such patients who move from place to place for a livelihood becomes extremely difficult. A good number of lost to follow-up cases belong to migrant workers in both districts. Children of such patients cannot be screened as they do not accompany their parents during migration.

*“Migrant patients take the treatment for one month. They are mostly from Bijapur and Gulbarga. They will go back to their place, and we cannot trace them as their phone numbers and address will not be correct…”*

(STS, Udupi district)

*“Bengaluru has a lot of migratory population if they leave this place then it becomes too challenging to monitor. When children go to their relative’s homes there are chances of missing the treatment…”*

(STS, Bengaluru district)

#### 3.1.2. Response to Screening

**1.**  ***Stigma and fear of disclosure:*** The healthcare providers shared that awareness of TB disease has garnered good attention in the urban area and that the rural area needs to evolve itself from stigmatizing the disease. Once the adults are diagnosed as TB patients, their children are immediately shifted to their relatives or neighboring houses. People possess the fear of disclosure as they fear being treated differently if others knew about their disease condition. Few patients ask the health staff to maintain confidentiality and the staff visit the patient’s household disguising as the patient’s relatives or friends, even a few patients have requested the health care staff not to visit their homes. Hence, screening pediatric contacts could be a missed opportunity. The health care providers generally convey information about the disease to the family in a sensitive manner based on the conduciveness and cultural context. People might face issues surrounding their relationships with regards to their jobs and marital conflict and end up losing their jobs and facing divorce.

*“We directly do not start talking about TB. We indirectly talk about it (disease) that time they get convinced. If we suddenly start talking about TB, then there are chances that the family might get divided. We talk to patients first; through patients, we talk to the family…”*

(TBHV, Udupi district)

*“According to our RNTCP, below 6 yrs we give prophylaxis but still there is a taboo. People will try to speak to us and tell ‘Sir we will shift the patient, we will say that no one was there in the house’ because they do not want to take the medication because of side effects…”*

(Medical officer, Udupi district)

*“For people who live in a rented house, the owner can be problematic. Same family members do not know about the patient having TB. If the wife has TB, then the husband does not know. If daughter-in-law has TB, then mother-in-law does not know…”*

(STS, Bengaluru district)

*“Some people ask us not to wear ID and if someone asks us then we tell we are the guests. These 2 things they restrict. Sometimes if the patient lives on the 2nd floor, then the patient comes down to meet us. In such cases, we ask the patient to come to the hospital…”*

(TBHV, Udupi district)

**2.**  ***Non-availability of family members***: Healthcare workers describe those patients who were less aware of the necessity of IPT and were more reluctant to participate in latent TB screening. Contact screening cannot be successfully achieved when all the members of the family are not present in the house during the visit of a healthcare provider for screening. Moreover, when the family is dependent on a person’s earnings, it is not feasible for that person to lose his earnings for the day. At times, patients might be the breadwinner of the family, and this compels them to continue working irrespective of their health conditions.

*“Not everyone will be present. If a house has 5 members, we might be able to meet 3 members only. We meet only ladies as gents go to work. There are times when we will not be able to meet TB patients as well. Kids will be there since it is corona now…”*

(STS, Udupi district)

**3.**  ***Healthcare staff not provided with due respect:*** The staff shared their experience of not being treated with due respect by the patients, especially if they have alcohol use disorders. Upon their visits, the health care providers were verbally abused by such patients and lack of co-operation further escalates the problem of screening the pediatric population at their houses.

*“When I was working in K.R. Puram, a patient was co-infected with TB. When I went to their home and told even his wife will have to get tested. He had come running to hit me with a metal rod…”*

(STS, Bengaluru district)

*“Chronic alcoholic patients are more in my TB unit. They scold us and try to hit us. They ask us not to come home…”*

(STS, Udupi district)

#### 3.1.3. Challenges Faced during the Investigation

**1.**  ***Challenges in the collection of samples—***As explained by the healthcare providers, sample collection in children is a complex procedure especially when gastric lavage must be obtained. The staff shared their experience that parents generally object to such procedures. This is one of the potential challenges that hinder the contact investigation procedure. Other additional factors are sparsely available skills and health infrastructure.

*“We can do CBNAAT procedure for Gastric lavage also but that is a difficult procedure because we must admit the child, we must do early morning aspiration. We cannot do it whenever we want. We should put a tube in the night and aspiration in the morning. If we put the tube in the morning, then secretion will move on to the duodenum. We will not get a proper sample that is a bit difficult procedure, Parents may not agree…”*

(Pediatrician, Udupi district)

**2.**  ***Choosing private facilities over Government—***Healthcare providers have witnessed parents being determined to provide the best of facilities for the well-being of their children in the notion that they opt for private healthcare facilities over the government. The follow-up of the child usually gets missed. The parents prefer to seek private health care services as they feel it is more secure and trustworthy since they believe that their health status is not revealed to the public health sector and hence, there are no follow-up visits by the health personnel. A private patient visits the hospital alone and does not take children to the hospital for evaluation and the opportunity for screening is missed.

*“In private cases, it is difficult to follow up. We get the data extremely late from the private sector sometimes after a month. Private patients ask us not to visit their homes or do not want government medicines…”*

(STS, Udupi district)

**3.**  ***Transportation issues to reach the hospital for investigation—***The non-availability of a well-established transportation system for the patients to commute to the healthcare facilities is a major drawback for evaluation as there is no equitable distribution of health facilities at most places. Moreover, the transportation cost is an out-of-pocket expenditure for the patient. Hence, people are hesitant to make multiple visits for investigations. It is desired to have a point of care diagnostic test for the pediatric population that can be conducted at the residence.

*“See from Kolluru they must come here and Maravanthe patients will have to go to Kundapura which is far. So, in between, if there is an X-ray facility it is better. If all the tests are done at one center it is more convenient rather than going to different places for different tests. At least one center with all the facilities every 20 km is better…”*

(STS, Udupi district)

*“It is challenging to get TST done. If a test is available in the hospital, then it is easier for the patient as well. If they will have to travel to different places for a different test, then investigations will be delayed…”*

(STS, Bengaluru district)

**4.**  ***Issue of the working hours—***The population willing to access the public health facility is high in bigger cities such as Bengaluru since it is provided free of cost. However, patients do not want to wait for a longer duration period owing to their work commitments and expect a time slot for their visit.

*“It is difficult to get the patient to the hospital. They ask us if the test is done for free, but if we must get a free test done then they do have to come at a specific time. Since parents work it gets delayed and they do not come when we ask them to come…”*

(TBHV, Bengaluru district)

#### 3.1.4. Treatment Initiation

**1.**  ***Anxiety due to long course of Chemoprophylaxis—***Chemoprophylaxis is given for 6 months which is the same as the duration of the patient’s treatment. Parents tend to worry when long courses of antibiotics are given.

*“If we say the child is positive, needs treatment they will be very anxious. That too when they see the tablet strips, they will be afraid to see so many drugs and big tablets. They will be worried since it must be given for six months. They will repeatedly ask if the child is positive? If the child needs so much treatment…”*

(Pediatrician, Udupi district)

**2.**  ***Delay in getting the tests done—***The healthcare staff noted a delay when parents spend money out of their pockets to get the investigations done in private labs. The suggested tests are freely available at select health facilities and many times the patients must shell out their money for such tests at private hospitals if these are not available. There remains a tendency among patients to delay such tests as they are not life-threatening. Bengaluru has a greater number of public and private health facilities in comparison with Udupi. While in Udupi, except the Udupi Taluka, other talukas are dependent mostly on public health facilities since there are only a few options of private health facilities.

*“Some people give economic reasons to get the investigations done. They will have to get CXR, TST outside so it might get delayed.* *…”*

(TBHV, Bengaluru district)

**3.**  ***Not visiting the hospital due to the fear of Coronavirus infection—***The onset of the COVID-19 pandemic has taken a toll over other infectious diseases since the focus has been shifted towards the COVID diagnosis and contact screening. People do not want to visit the hospital in the fear of contracting infections.

*“People neglect the treatment or do not want to come to the hospital now as they have the fear of Corona. Earlier the problem was not being aware…”*

(ASHA, Udupi district)

#### 3.1.5. Supervision and Monitoring

**1.**  ***Lack of adherence record maintenance for Chemoprophylaxis—***Adherence is stringently monitored only in the case of the patient but not in the case of a child administered with IPT. Hence, there are chances of missing doses.

*“Adherence is not maintained in the Nikshay application* (a web-based online portal for TB notification and patient care management in India)*. We will just mention the date when the medicines were given…”*

(STS, Bengaluru district)

### 3.2. Suggestions

**Figure 3:**
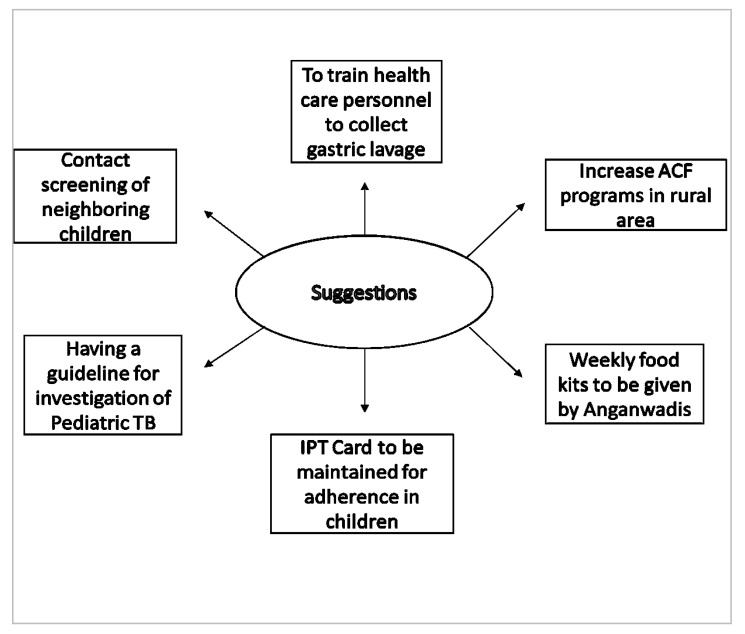
Depicting the key suggestions to improve contact screening and IPT initiation in children.

The suggestions from the health care workers were mainly (1) to build the capacity of the health care personnel in obtaining the gastric lavage; (2) augment the active case finding activities in the rural area; (3) provision of nutritious food; (4) maintenance of IPT card for treatment monitoring; (5) standard protocol to guide the pediatric TB investigation; (6) contact screening of children in the neighborhood. The key suggestions by the healthcare staff to improve contact screening and IPT initiation in children are described in Figure 3.

1. Evidence-based diagnosis is important for confirmation of pediatric TB. In practice, gastric lavage is being collected in pediatric age groups for which the child must be admitted to the hospital overnight but, in most cases, parents disagree with such elaborate procedures. As per the recently revised guidelines, collection of induced sputum is recommended. With consistent training for the practitioner at the peripheral level, challenges concerning sample collection can be resolved.

*“There is little difficulty at implementation level mainly because gastric lavage is a challenging procedure, but revised guideline has a collection of induced sputum. But our public pediatricians are not yet trained for it. Even in the private sector, the diagnosis of pediatric TB is done clinically. Less importance is given to microbiological confirmation by obtaining a sputum sample. This is a major drawback both in the public and private sectors. We must tell them about the importance frequently. One fine day all the guidelines will be implemented…“*

(DTO)

2. Health workers describe that the awareness of various programmes must be given on various programmes and benefits under the government. Educating people about the disease and the symptoms will lead to better enrolment thereby early diagnosis.

*“IEC should be there. We must guide them and educate them about the symptoms of HIV and TB. Awareness regarding every disease. Symptoms and spread of TB should be known. All the patients should be referred to the government. Now for TB, leprosy, and HIV, we have the best medicines here. The main thing is awareness about these diseases and facilities given by the government. Rural people should know. Automatically they will come. They will spread the word and guide other people also…”*

(Medical officer, Udupi district)

3. The NTEP is providing an incentive of nearly USD 7 per month as support for nutritious food under Nikshay Poshan Yojana for all the patients under the programme through a direct benefit transfer. Few patients have the opinion that rather than providing monetary support, nutritious food could have been distributed to patients and their families.

*“Packets of protein-rich food can be provided. Perishables like milk and egg we cannot do anything, but protein-rich food provided by taluka level will help. Because Rs.500 they may spend it before going home also and some will have the habit of alcoholism. Usually, this disease is more prevalent in low socioeconomic status people so seeing that money they may use for something else also…”*

(Pediatrician, Udupi district)

4. Under the NTEP, only patient treatment adherence is maintained. Adherence to IPT can also be maintained for children. It can be done by updating the Nikshay application or by providing a treatment card.

*“If a card is maintained for taking INH also then it will be more helpful…”*

(STS, Udupi district)

5. Children less than 6 years are examined for symptoms and investigated, while the age group above 6 years is only examined for symptoms. A systematic guideline based on the age criteria and latency of the disease can prevent the occurrence of the disease.

*“We do not have a guideline for screening latent TB yet. For children above 6 years and adults, we can try to find out latent TB infection if included in the programme. Once we have guidelines for latent TB infection then everyone will be included, and evaluation can be carried out. For now, we do not have such guidelines. For children above 6 years old, they will have symptoms but one symptom, that is neglected is weight loss or no gain in weight by our staff. Weight loss is also one of the signs of TB not always pediatric contact presents with fever and cough. Because our staff will be mainly concentrating only on pediatric contact less than 6. This is the main problem in the programme concerning pediatric contact. For more than 6 years of age, we treat like other contacts which should not happen in my opinion…”*

(DTO)

6. In India, houses are placed closely, and the families have constant interaction with each other. Hence, it is important to screen not only the children living in the same household but also children living in the neighborhood as they are also in constant contact with the TB patient. Few of the healthcare professionals suggest that screening of neighboring children will certainly be helpful in the prevention of the disease which might otherwise appear in the future.

*“I think one belt of surrounding house children also must be sent for contact screening, that should be improved…”*

(Medical officer, Udupi district)

## 4. Discussion

This study is one of the few studies conducted in recent years to identify the challenges for contact screening and initiation of IPT chemoprophylaxis among child contacts. Our findings suggest that operationally there are magnanimous challenges in the field that the programme must holistically address. Broadly, the challenges are related to reaching out to patients, stigma towards the disease, getting them screened, and addressing their concerns for investigations. The patients would have made an opinion before the health care workers reach out to them for child contact screening. A qualitative study carried out in Lesotho faced a similar challenge such as cost transportation due to the long distance to clinics from remote areas and stigma as the key challenge in pediatric contact investigation [9]. A study carried out by Belgaumber et al. also suggested that stigma-related disclosure is one of the predominant barriers faced in pediatric contact screening [15]. Extensive education through electronic media and innovative campaigns involving schools as well as non-governmental organizations could be the best strategy to approach the masses. A massive campaign similar to “Swacch Bharath”, which is a massive mass movement that seeks to create a Clean India, should be undertaken by the country to completely erase the stigmatic issues related to the disease such as TB, HIV, Leprosy, etc. Supervision and monitoring of patients on IPT is a challenge even now. Though many studies have consistently identified the lacunae, no innovative mechanisms are in place by the programme. A study by Singh et al. carried out in Bhopal (central India) concluded similar findings as our study such as poor monitoring of IPT initiation and lack of awareness as the major barrier in the implementation of contact screening [16]. A study in 2011 by Pothukuchi et al. concluded that rigorous monitoring and awareness of the contact screening and IPT provision will assure better implementation of the programme [10]. A study conducted by Banu Rekha et al. interviewed 253 PTB patients and found that documentation of the treatment was lacking, and people were not aware of the detailed procedure of contact screening [17]. A study conducted by Sivaramakrishna et al. revealed that the IPT uptake was irregular and adequate education was not given to people [18]. The programme may consider opening a separate wing at central, state, and district TB centers augmented with adequate human resources for monitoring of child contact screening and IPT in the field.

The recommendations from the health care providers are summarized in the Figure 3. Few healthcare providers shared valuable suggestions to help in the betterment of the programme. Training the health personnel to collect the sample will make the diagnosis of pediatric TB more sensitive and specific. Increasing ACF programmes and IEC programmes will resolve the stigma against these communicable diseases. Nikshay Poshan Yojana in India was started in 2018, where the patient is given INR 500 for consumption of nutritious food but in some cases that benefit was being used for various other purposes [19]. The health workers recommended utilizing that fund to indirectly provide the patients with nutrition-rich food to the patient and their families. Having maintained an IPT adherence card will help in tracing the treatment for the contacts, as well. A standardized guideline should be made to follow pan-India for all the investigation processes of pediatric contacts. TB patients come in contact with the neighboring children on a regular basis, hence the field staff can screen for any symptoms related to TB.

This study will help to identify the gaps between policy and practice. Our study has a few strengths and weaknesses. The strengths are: (1) The study has explored the various reasons for inadequate implementation of screening process and difficulties in institution of chemoprophylaxis. (2) The study has interviewed the staff at the different levels under NTEP, hence challenges at individual levels were identified which are unique. (3) Two districts that are ecologically and socially different were selected to compare the challenges. (4) An attempt is made to include suggestions from the health care workers who are actively involved in the implementation of the programme at grass-root levels, and it may provide insights to the policymakers for better implementation.

The limitations are: (1) In the process of data collection, the key informant-based interview was conducted. In a couple of situations, the health care workers faced difficulties in understanding the questions posed to them. We are afraid that it may or may not lead to minimal bias. (2) Since the interviews were recorded, some staff might have refrained from revealing their exact opinion. (3) The findings of our study cannot be generalized for other districts. However, the policymakers can incorporate the key recommendations and implement them in the programme accordingly.

## 5. Conclusions

Overall, there are universal implementational challenges that include reaching out to the patients, stigma towards the disease, getting the child contacts screened, and addressing parents’ concerns for investigations. It is imperative for the programme to address these challenges efficiently and effectively. The programme must embark on innovative solutions, feasible engagements, and massive efforts to improve the uptake and prioritize the monitoring of contact screening and IPT administration.

## Data Availability

The data presented in this study are available on request from the corresponding author. The data are not publicly available due to privacy and ethical considerations.
